# Cross‐Cultural Adaptation and Validation of the Organizational Readiness for Implementing Change (ORIC) Scale Among Nurses in Chinese Healthcare Institutions: A Multilevel Psychometric Assessment

**DOI:** 10.1155/jonm/2913316

**Published:** 2026-08-12

**Authors:** Yu Tian, Ling Yu, Hui Wang, Yanze Shi, Lei Liu, Yingjie Wang, Zihan Li

**Affiliations:** ^1^ Nursing Department, Cancer Hospital of Dalian University of Technology (Liaoning Cancer Hospital & Institute), Shenyang, China; ^2^ Department of Thoracic Surgery, Cancer Hospital of Dalian University of Technology (Liaoning Cancer Hospital & Institute), Shenyang, China; ^3^ School of Nursing, Liaoning University of Traditional Chinese Medicine, Shenyang, China, lnutcm.edu.cn; ^4^ Nursing Department, The First Affiliated Hospital of Liaoning University of Traditional Chinese Medicine, Shenyang, China

**Keywords:** organizational readiness, ORIC, psychometrics, reliability, validity

## Abstract

**Background:**

Nurses are increasingly required to adapt to organizational changes related to healthcare reform. Organizational members’ readiness for implementing change is a key determinant of effective change. However, a reliable tool for assessing organizational readiness for implementing change among Chinese nurses is currently lacking.

**Aims:**

To cross‐culturally adapt the Organizational Readiness for Implementing Change (ORIC) scale and validate its psychometric properties in the context of Chinese healthcare institutions.

**Methods:**

In this cross‐sectional study, Samples A and B were collected using convenience and stratified convenience sampling from four tertiary Grade A hospitals from July to December 2025. Data were collected using the General Information Questionnaire and the adapted Chinese ORIC scale. Sample A was used for item analysis and exploratory factor analysis, while Sample B was used for multilevel confirmatory factor analysis and multilevel reliability to examine the psychometric properties of the ORIC scale.

**Results:**

The valid response rates were 100% for Sample A and 93.33% for Sample B. Item analysis supported the retention of all 10 items (*r* > 0.300, *p* < 0.050). Exploratory factor analysis extracted two factors, accounting for 79.81% of the total variance. Multilevel confirmatory factor analysis demonstrated a good fit of the two‐factor structure at the organizational level (*χ* ^2^/df = 1.211, RMSEA = 0.014, CFI = 0.978, TLI = 0.988, SRMR‐Within = 0.024, SRMR‐Between = 0.017). Reliability analyses indicated good multilevel internal consistency (Cronbach’s ɑ = 0.771–0.988) and satisfactory test–retest reliability (*p* < 0.001).

**Conclusions:**

The Chinese ORIC scale comprises two dimensions with 10 items. Following rigorous cross‐cultural adaptation and validation, it demonstrates good psychometric properties and can be used to assess nurses’ organizational readiness for implementing change in Chinese healthcare institutions.

**Implications for Nursing Management:**

The validated Chinese ORIC scale provides nursing managers with an evidence‐based tool to assess organizational readiness for implementing change from the perspective of nurses as organizational members. By capturing shared perceptions of change commitment and change efficacy at the organizational level, it can inform targeted implementation strategies, strengthen organizational support, and facilitate successful healthcare change initiatives.

## 1. Introduction

Global healthcare organizations are currently undergoing major transformation driven by digitalization, multidisciplinary collaboration, and the widespread implementation of precision medicine, with the aim of improving the quality of healthcare services and addressing increasingly complex public health challenges [1, 2]. Simultaneously, China is actively implementing healthcare change, aiming to optimize resource allocation and promote equity and accessibility in healthcare, thereby ultimately enhancing public health and ensuring innovative and sustainable development of healthcare systems [3]. Despite significant achievements, rapid and continuous processes of organizational change often entail organizational challenges [4, 5]. Research has consistently demonstrated that sufficient organizational readiness for change (ORC) is a crucial prerequisite for successful implementation of new policies, technologies, and innovative projects [6–8]. ORC not only facilitates effective translation and implementation of change initiatives but also supports successful execution of complex organizational change within healthcare settings [6–8]. Conversely, insufficient ORC may lead to adverse outcomes such as change fatigue, increased employee stress, and reduced job satisfaction, and may even compromise intended outcomes of change initiatives [9]. Therefore, adequate ORC is essential for ensuring sustainable implementation of change initiatives.

ORC is defined as the degree to which members within an organization are psychologically and behaviorally prepared for implementing organizational change [10]. Grounded in Weiner’s theory, ORC is conceptualized as a multidimensional construct comprising two core dimensions: change commitment and change efficacy [7]. Change commitment refers to members’ shared resolve and willingness to implement change, whereas change efficacy reflects collective beliefs in the organization’s capability to execute the change, including perceived task knowledge, resources, and situational support [7]. Importantly, ORC is conceptually distinct from several closely related constructs that are often conflated in organizational research. Specifically, organizational culture refers to deeply embedded and relatively stable values, beliefs, and assumptions that characterize an organization over time [11], whereas ORC is a change‐specific and temporally sensitive state reflecting readiness for a particular initiative [10]. Organizational climate captures shared perceptions of general organizational practices and work environment [12], whereas ORC is explicitly directed toward a specific organizational change effort [10]. In contrast, individual change intention is an attitudinal and motivational disposition at the personal level [13], whereas ORC is an emergent collective construct formed through social interaction and shared sense making processes [10]. Therefore, ORC cannot be adequately represented by simple aggregation of individual attitudes. Instead, it emerges at the organizational level and should be examined with appropriate assessment of aggregation validity to ensure construct validity.

Research has shown that systematically assessing ORC helps identify potential obstacles during change implementation and informs the development of effective intervention strategies [14]. However, existing research exhibits several limitations. Firstly, many studies rely on simple aggregation of individual‐level responses to represent organizational constructs, without sufficient examination of multilevel measurement properties, which may compromise the validity of organizational‐level inferences [14–17]. Secondly, some instruments were developed without an articulated theoretical framework, resulting in limited evidence supporting their psychometric robustness [10, 18, 19]. Finally, although some studies have demonstrated acceptable psychometric properties, the large number of items included in these instruments may limit their feasibility in busy healthcare settings [16]. Taken together, these limitations highlight the need for a theoretically grounded, psychometrically robust, and practically feasible instrument capable of accurately capturing ORC in healthcare contexts.

Grounded in Weiner’s theory of ORC, the Organizational Readiness for Implementing Change (ORIC) scale was developed by Shea et al. in 2014 [20]. By assessing the core constructs of change commitment and change efficacy at the organizational level, the ORIC scale extends beyond individual‐level perspectives and addresses limitations of earlier individual‐focused approaches [20]. Furthermore, its concise structure and practical applicability make it particularly suitable for use in complex healthcare settings [20]. The ORIC scale has subsequently been translated into several languages, with studies consistently supporting its psychometric properties across diverse cultural contexts [17, 21–23]. Cross‐cultural adaptation of the ORIC scale requires not only linguistic translation but also ensuring conceptual and contextual equivalence in the target setting, as interpretations of change commitment and change efficacy may vary across healthcare systems [7, 20]. Although the ORIC scale has been widely used internationally, a formally adapted and psychometrically validated Chinese version for healthcare organizations is still lacking. This gap limits the assessment of organizational‐level readiness for change in Chinese healthcare settings and highlights the need for rigorous cross‐cultural adaptation and multilevel validation. In Chinese healthcare organizations, change is typically implemented within hierarchical structures characterized by centralized decision‐making and top‐down policy implementation [24]. In this context, change commitment is more likely to reflect shared willingness to engage in organizational change, whereas change efficacy is shaped by collective perceptions of organizational capability, resources, and support [7, 20]. These contextual features may influence how ORIC items are interpreted, further emphasizing the importance of establishing measurement equivalence.

Accordingly, this study aims to conduct a cross‐cultural adaptation and psychometric evaluation of the ORIC scale in Chinese healthcare settings, including assessment of content validity, structural validity using exploratory and multilevel confirmatory factor analysis (MCFA), and reliability in terms of internal consistency and test–retest stability. This study seeks to provide a culturally appropriate instrument for measuring ORC and extend evidence of its applicability in non‐Western healthcare contexts.

## 2. Methods

### 2.1. Scale Introduction

Developed and validated within healthcare organizations, the ORIC scale assesses two core dimensions: change commitment (items 1–5), which captures employees’ collective willingness to change, and change efficacy (items 6–10), which measures their collective confidence in implementing change successfully [20]. The scale employs a five‐point Likert scale ranging from 1 (“strongly disagree”) to 5 (“strongly agree”) for all items [20]. The original validation study confirmed its strong psychometric properties, reporting a Cronbach’s α of 0.880 and good structural validity. Moreover, high aggregation reliability was demonstrated under controlled laboratory conditions (Interclass Correlation Coefficient, ICC = 0.720–0.980) [20].

### 2.2. Study Design and Process

This cross‐sectional study included two independent samples. Sample A was obtained using convenience sampling from one tertiary Grade A hospital. Sample B was collected from three tertiary Grade A hospitals using a stratified convenience sampling method, with hospitals serving as the primary strata and nursing units within each hospital serving as secondary strata. Eligible nurses were collected from each unit. Data collection was conducted from July to December 2025.

**FIGURE 1:**
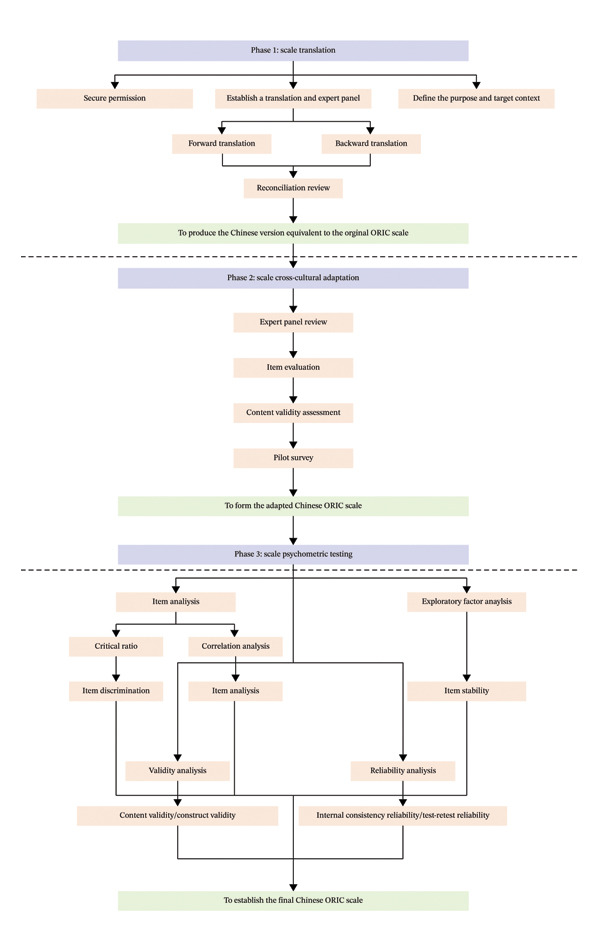
Flowchart of the cross‐cultural adaptation and validation of the organizational readiness for implementing change scale. Created using Microsoft PowerPoint 2019 (Microsoft Corporation, Redmond, WA, USA).

A multiphase methodological approach was employed to validate the original ORIC scale within the Chinese healthcare context. This process involved three main phases: (1) Scale translation: a forward–backward translation procedure was conducted to produce the Chinese version equivalent to the original ORIC scale. (2) Scale cross‐cultural adaptation: The Chinese version equivalent to the original ORIC scale was preliminarily evaluated and refined to ensure its cultural relevance and feasibility in the Chinese context, thereby forming the adapted Chinese ORIC scale. (3) Scale psychometric testing: The adapted Chinese version of the ORIC scale was tested to systematically assess the psychometric properties, thereby establishing the final Chinese ORIC scale. The detailed procedures are depicted in Figure 1.

### 2.3. Subjects

Since subspecialties (e.g., cardiology and neurology) often operate under homogeneous management structures and cannot adequately capture interdepartmental differences, this study selected large frontline clinical departments (e.g., internal medicine and surgery) as sampling units. Data were then collected from nurses within these departments who met the study criteria. Focusing on nurses is justified by their central role in implementing change in clinical settings, making them crucial for studying change‐related constructs [25]. Inclusion criteria: (1) registered nurses who had worked in the corresponding organization for at least 6 months; (2) registered nurses who were in active service during the survey period; (3) registered nurses who voluntarily agreed to participate in this study; and (4) registered nurses who were able to independently comprehend and complete the questionnaire. Exclusion criteria: (1) individuals who were nursing interns, visiting nurses, or employees who had been absent from duty for an extended period (individual level) and (2) organizations with fewer than 5 participants, as they could not meet the requirements for organizational‐level analysis (organizational level) [26].

### 2.4. Sample Size

Sample size determination accounted for the distinct requirements of various statistical analyses. (1) Exploratory factor analysis (EFA): A minimum sample size of 100 participants is required for EFA [27]. Considering an anticipated attrition rate of 20.00%, at least 120 participants were necessary (Sample A). (2) MCFA: For MCFA, a minimum of 51 units at the organizational level and at least 5 members per unit at the individual level are required [26]. To account for a 20.00% inefficiency rate, at least 319 participants from 64 departments (Sample B) are required. (3) Multilevel test–retest reliability: From Sample B, 200 participants were included in the test–retest reliability analysis [28]. The final sample size was chosen to satisfy the most demanding criteria across all analyses. In Sample A, all 120 distributed questionnaires were returned and valid (100.00% response and validity rates). In Sample B, of 1200 questionnaires distributed, 1146 were returned and 1120 were valid, yielding response and validity rates of 95.50% and 93.33%, respectively.

### 2.5. Scale Translation

In November 2023, authorization was obtained by contacting the corresponding author via email. A translation team was subsequently formed in March 2025, and the ORIC scale was culturally adapted into Chinese following the Brislin translation model [29]. The detailed procedure is as follows: (1) Forward translation: experts F1 and F2 independently performed the forward translation. (2) Review of forward translation: experts R1 and R2 reviewed and discussed the results of forward translation to reach a consensus version. (3) Back translation: experts B1 and B2, who had no prior exposure to the ORIC and were not involved in the forward translation, independently performed the back translation. (4) Review of back translation: experts R3 and R4 reviewed and discussed the results of back translation to produce the Chinese version equivalent to the original ORIC scale.

During the forward translation process, minor discrepancies were identified in the translation of key concepts, item wording, personal pronouns, and semantic nuances. For example, alternative translations were proposed for terms such as “change” and “committed to”. These discrepancies were resolved through discussion and consensus among proofreaders R1 and R2, with decisions guided by conceptual equivalence, semantic accuracy, linguistic clarity, and cultural appropriateness. Similar procedures were followed during the back‐translation review stage. Detailed examples of the translation and adaptation process are provided in Appendix Table A2.

### 2.6. Scale Cross‐Cultural Adaptation

To evaluate the content validity of the Chinese equivalent of the original ORIC scale, the scale was revised by a panel of eight experts with extensive relevant experience, none of whom had been involved in its initial translation. The expert panel rated the relevance of each item to its corresponding dimension using a 4‐point ordinal scale (1 = not relevant, 4 = highly relevant) [30]. Ratings of 1 or 2 were considered “not relevant”, whereas ratings of 3 or 4 were considered “relevant” [30]. The item‐level content validity index (I‐CVI) was calculated as the proportion of experts rating an item as relevant (3 or 4), using the formula: I‐CVI = *n*_*e*_/*N*, where *n*_*e*_ is the number of experts assigning a rating of 3 or 4, and *N* is the total number of experts [30]. The S‐CVI was calculated as the average of I‐CVI values across all items: S‐CVI = (∑I‐CVI_*i*_)/*k*, where *k* denotes the total number of items [30]. Following established thresholds, items were retained if I‐CVI ≥ 0.800, and the overall scale was considered valid if S‐CVI ≥ 0.900 [30].

The expert panel consisted of members with diverse professional backgrounds, including nursing management, implementation science, and English linguistics. Several experts also had international educational experience, and their professional experience ranged from 10 to 20 years. Detailed characteristics are presented in Appendix Table 1.

### 2.7. Pilot Survey

A pilot survey was conducted with 30 participants to evaluate the clarity and comprehensibility of the adapted Chinese ORIC scale. The primary objective was to finalize the adaptation by ensuring the translated items were linguistically and conceptually equivalent to the original. Participants completed the questionnaire and provided feedback on the item wording, response options, and overall comprehension.

### 2.8. Instruments

#### 2.8.1. General Information Questionnaire

Informed by a literature review and experts’ consultation, we developed a Sociodemographic Characteristics Questionnaire that comprised the following variables: age, gender, educational level, marital status, professional title, professional qualification, managerial positions, and work schedule type.

#### 2.8.2. The Adapted Chinese ORIC Scale

After translation and cross‐cultural adaptation, the adapted Chinese ORIC scale was developed. A 5‐point Likert scale ranging from 1 (“strongly disagree”) to 5 (“strongly agree”). The higher the score, the greater the ORC among organizational members.

### 2.9. Data Collection

An online questionnaire was administered via Wenjuanxing (https://www.wjx.cn), a widely used and reputable survey platform in China, to facilitate efficient and standardized data collection. An anonymous link was distributed through official hospital communication channels (e.g., department WeChat groups) for participants to access. All data collected from the platform were downloaded, imported into Excel 2016 (Microsoft Corp., Redmond, WA, USA), and then independently verified by two investigators to ensure validity prior to statistical analysis.

### 2.10. Quality Control

To ensure data quality, a pilot survey was conducted to assess item clarity and comprehensibility. All questionnaires were meticulously reviewed to prevent missing or inconsistent data, with follow‐up confirmation performed when necessary. To minimize investigator‐related bias, all research staff received standardized training prior to data collection. Furthermore, in determining the sample size, a margin of error of 20.00% was incorporated to partially mitigate potential bias resulting from the use of convenience sampling.

### 2.11. Statistical Analyses

Statistical analyses were performed using SPSS Version 25 (IBM Corp., Armonk, NY, USA) and Mplus version 8.11 (Muthén and Muthén, Los Angeles, CA, USA). All hypothesis tests were two‐tailed, with a significance level set at 0.050.

(1) Descriptive statistical analysis: categorical variables were expressed as frequencies (%), normally distributed continuous variables were presented as mean ± standard deviation (mean ± SD). (2) Item analysis: Item analysis was evaluated using two complementary methods based on data of Sample A. The extreme group method, which was applied to assess the overall quality of the scale, and the correlation coefficient method, which was used to evaluate the quality of each individual item. In the extreme group method, the total scores of the scale were ranked from highest to lowest, and independent‐samples t‐tests were performed between the top 27.00% and the bottom 27.00% of respondents [31]. A *p* ≤ 0.050 indicated the differences between the two groups on each item were statistically significant. In the correlation coefficient method, items with *r* > 0.300 and *p* ≤ 0.050 were considered to have good discriminative validity [31]. (3) EFA: EFA was conducted using data from Sample A. Kaiser–Meyer–Olkin (KMO) value > 0.800 indicated that the data were suitable for EFA [32]. Cumulative variance contribution rate of the common factors greater than 40.00% and factor loadings of each item on its corresponding factor greater than 0.400 suggested that the extracted factors adequately reflected the information of their respective dimensions [32]. (4) MCFA: The structural validity of the adapted Chinese ORIC scale was examined using data from Sample B through Muthén’s “five‐step MCFA approach” [33], which provides a systematic framework to address model complexity and convergence issues by sequentially conducting traditional CFA, estimating within‐ and between‐group components, and final MCFA, with each step building upon the previous. The MCFA consists of five steps: traditional CFA, estimation of between‐group variance, estimation of within‐group structure, estimation of between‐group structure, and MCFA [33]. Model fit was considered acceptable when the following criteria were met [34]: Chi‐square/degree of Freedom (*χ* ^2^/df) < 3, Root Mean Square Error of Approximation (RMSEA) ≤ 0.080, Comparative Fit Index (CFI) > 0.900, Tucker–Lewis index (TLI) > 0.900, Standardized Root Mean Square Residual (SRMR) < 0.100. Differently, the ICC and the design effect were used as reference indicators in estimating between‐group variance, with an ICC ≥ 0.059 and a design effect > 2 indicating substantial between‐group differences for each item, and the absence of such differences suggesting that subsequent multilevel modeling was unnecessary [33]. ICC ≥ 0.059 was adopted as an index of clustering to assess between‐group variance in the MCFA procedure, rather than as a reliability or stability coefficient [33]. (5) Multilevel reliability analysis: The reliability of the adapted Chinese ORIC scale was evaluated in terms of both internal consistency and multilevel test–retest reliability. Internal consistency was assessed using the multilevel Cronbach’s ɑ, with values greater than 0.800 for the overall scale and greater than 0.700 for each factor considered indicative of good reliability [35]. Multilevel test–retest reliability was examined after a 2 week interval among 200 participants from Sample B, using the Intraclass Correlation Coefficient (ICC), with a value above 0.750 regarded as indicative of good temporal stability [36].

### 2.12. Ethical Consideration

Prior to participating in this study, all participants provided informed consent, which detailed the study’s objectives, procedures, potential risks and benefits, and their right to withdraw at any time without negative consequences. To protect participants’ privacy, all personally identifiable information was excluded from the dataset upon collection. The data were securely stored, with access restricted solely to authorized researchers. This study was conducted in accordance with the Declaration of Helsinki (24th version). The study was reviewed and approved by the Medical Research Ethics Committee of the Liaoning Cancer Hospital (Approval No.: KY20241103).

## 3. Results

### 3.1. General Demographic Characteristics of the Participants

A total of 120 participants were included in Sample A, consisting of 14 males and 106 females, aged 23–57 years. Among them, 117 (97.49%) had attained an undergraduate education or above, while only 3 (2.50%) held qualifications from junior college. In terms of professional profile, 13 (10.83%) held senior professional titles, and 11 (9.16%) occupied managerial positions. Shift work was the most prevalent work schedule, accounting for 51.66% of the sample A. For Sample B, 1120 eligible participants aged 23–55 years were collected, with general demographic characteristics largely similar to those of Sample A. Detailed information is presented in Table 1.

**TABLE 1:** Sociodemographic characteristics of the participants.

Variables	Group	Sample A (*n* = 120)	Sample B (*n* = 1120)
		*n* (%)/Mean ± SD	*n* (%)/Mean ± SD
Age		35.02 ± 7.765	33.08 ± 5.039

Gender	Male	14 (11.66)	106 (9.46)
	Female	106 (88.33)	1014 (90.53)

Educational level	Junior college or below	3 (2.50)	36 (3.21)
	Undergraduate degree	110 (91.66)	1031 (92.05)
	Graduate degree or above	7 (5.83)	53 (4.73)

Marital status	Unmarried	33 (27.50)	473 (42.23)
	Married	81 (67.50)	626 (55.89)
	Divorced/widowed	6 (5.00)	21 (1.87)

Professional title	Primary	43 (35.83)	434 (38.75)
	Intermediate	64 (53.33)	629 (56.16)
	Senior	13 (10.83)	57 (5.08)

Professional qualification	Yes	11 (9.16)	170 (15.17)
	No	109 (90.83)	950 (84.82)

Managerial positions	Yes	8 (6.66)	31 (2.76)
	No	112 (93.33)	1089 (97.23)

Work schedule type	Fixed day shift	36 (30.00)	436 (38.92)
	Shift work	62 (51.66)	557 (49.73)
	Fixed night shift	4 (3.33)	51 (4.55)
	Flexible work	18 (15.00)	76 (6.78)

### 3.2. Response Distribution of the Adapted Chinese ORIC Scale

**FIGURE 2:**
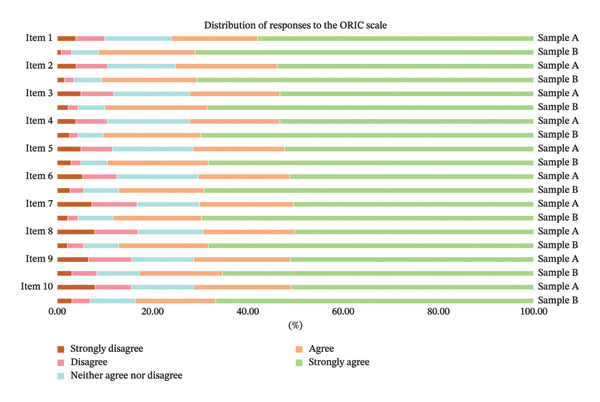
Response distribution of the adapted Chinese organizational readiness for implementing change scale. Created using Microsoft PowerPoint 2019 (Microsoft Corporation, Redmond, WA, USA).

In Sample A, the overall mean score of the adapted Chinese ORIC scale was 44.130 (item mean score = 4.410), while in Sample B was 46.69 (4.670). Overall, the proportion of respondents who selected “agree” and “strongly agree” was lower for the Change Efficacy dimension than for the Change Commitment dimension (86.22% < 91.50%). For example, the agreement rate for the item “Will do whatever it takes to implement this change” was notably higher than that for “Feel confident that they can handle the challenges that might arise in implementing this change” (92.41% > 83.66%). Detailed response distributions are presented in Figure 2.

### 3.3. Scale Translation and Cross‐Cultural Adaptation

The translation team developed the Chinese version equivalent to the original ORIC scale, as summarized in Appendix Table 2. An expert panel was then invited to evaluate the content validity of the scale and its items. All questionnaires were returned with a 100% recovery rate. The value of I‐CVI ranged from 0.830 to 1.000, and S‐CVI was 0.960; further details are provided in Appendix Table 3. In addition to the quantitative assessment, the experts participated in‐depth discussions regarding the cultural adaptation of the Chinese translation. The detail revision process based on these discussions is documented in Appendix Table 3.

### 3.4. Pilot Survey

A pilot survey was conducted with 30 participants. All of them completed the questionnaire within 6 min, indicating that the scale items were clearly expressed, easy to understand, and feasible for use in the busy healthcare context.

### 3.5. Item Evaluation and Preliminary Exploration

Item analysis was performed on data from Sample A to evaluate the discriminative power and inform item retention for the adapted Chinese ORIC scale. Using the extreme group method, participants were divided into a high‐scoring group (top 27%, total score ≥ 35, *n* = 38) and a low‐scoring group (bottom 27%, total score ≤ 30, *n* = 35). Independent‐samples t‐tests confirmed that all items exhibited statistically significant discriminative power, with *p* < 0.001 and 95% confidence intervals for the mean differences excluding 0. Results of the correlation coefficient method indicated that all 10 items demonstrated acceptable correlations with the total score (*r* > 0.300, *p* < 0.050). Therefore, all 10 items were retained for subsequent analyses.

**FIGURE 3:**
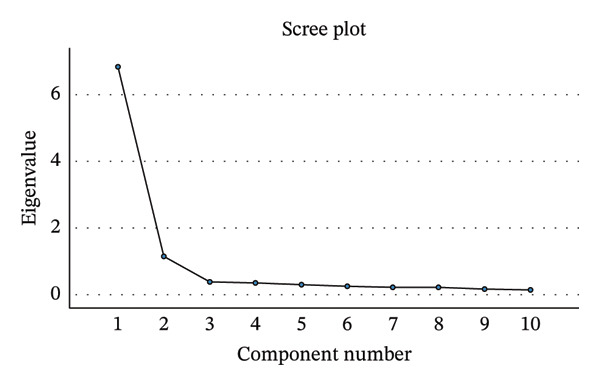
Scree plot for the exploratory factor analysis of the adapted Chinese organizational readiness for implementing change scale. Created using SPSS Version 25 (IBM Corp., Armonk, NY, USA).

EFA was conducted to examine the internal structure of the adapted Chinese ORIC scale. The data were deemed suitable for EFA, as indicated by a KMO value of 0.937. The scree plot (Figure 3) showed a distinct break after the second component, supporting the extraction of two factors. These two factors collectively accounted for 79.81% of the total variance, with all 10 items loading highly on their respective factors (range from 0.742 to 0.872). The final factor structure retained all original items and aligned with the two‐factor framework of the source version. Detailed results are presented in Table 2.

**TABLE 2:** Item analysis, exploratory factor analysis, and multilevel confirmatory factor analysis (Step 2) results of the adapted Chinese ORIC scale.

Factor	Item	Extreme group method	Correlation coefficient method	EFA	MCFA‐step 2	
		*t*	*r*	Factor loading	ICC	Effect size threshold
Factor 1	Item 1	−3.473^∗^	0.356^∗∗^	0.799	0.422	9.018
	Item 2	−5.258^∗∗^	0.456^∗∗^	0.872	0.387	8.353
	Item 3	−3.432^∗^	0.400^∗∗^	0.817	0.375	8.125
	Item 4	−3.953^∗∗^	0.378^∗∗^	0.824	0.388	8.372
	Item 5	−4.522^∗∗^	0.428^∗∗^	0.844	0.303	6.757

Factor 2	Item 6	−2.555^∗^	0.243^∗∗^	0.818	0.287	6.453
	Item 7	−4.045^∗∗^	0.376^∗∗^	0.873	0.324	7.156
	Item 8	−2.422^∗^	0.193^∗^	0.742	0.249	5.731
	Item 9	−2.879^∗^	0.242^∗∗^	0.786	0.264	6.016
	Item 10	−2.188^∗^	0.229^∗^	0.821	0.297	6.643

*Note:* Effect size threshold = 1 + (team size − 1) ∗ ICC value.

Abbreviation: ICC, Intraclass Correlation Coefficient.

^∗^ *p* < 0.05.

^∗∗^ *p* < 0.001.

### 3.6. Assessment of the Psychometric Properties of the Adapted ORIC Scale

#### 3.6.1. MCFA

**FIGURE 4:**
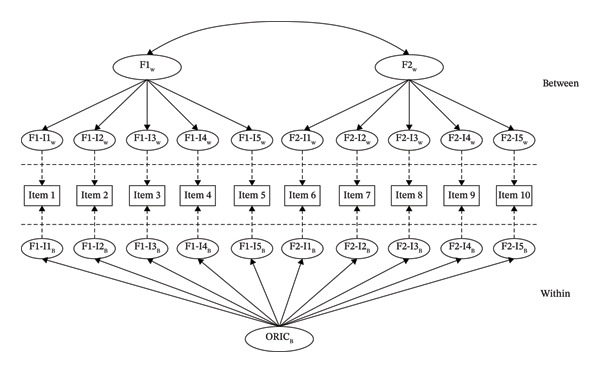
Structural diagram of multilevel confirmatory factor analysis for the adapted Chinese organizational readiness for implementing change scale. Created using Mplus version 8.11 (Muthén and Muthén, Los Angeles, CA, USA). Note: F = factor; I = item; W = within level; B = between level (e.g., F1‐I1_W_ denotes Item1 of Factor1 at the within level).

(1) Traditional CFA: The initial model showed inadequate fit (*χ* ^2^/df = 45.937, RMSEA = 0.137, CFI = 0.587, TLI = 0.530, SRMR = 0.571), indicating the need to examine potential organizational‐level differences (see Table 3 for details). The poor fit of the traditional CFA model is likely attributable to the nested data structure of participants within organizations, violating the independence assumption. Unmodeled clustering effects may inflate residual variance and bias parameter estimates, thereby reducing model fit indices, rather than indicating misspecification of the hypothesized two‐factor structure. (2) Estimation of Between‐Group Variance: ICC values for each item ranged from 0.249 to 0.422, with effect sizes between 5.761 and 9.018, demonstrating substantial between‐group variance across all items and justifying the use of multilevel analysis (see Table 2 for details). (3) Estimation of Within‐Group Structure: The within‐group model fit was unsatisfactory (*χ* ^2^/df = 25.601, RMSEA = 0.663, CFI = 0.698, TLI = 0.600, SRMR = 0.035), suggesting that the factor structure may not be consistent across groups and supporting the need for MCFA to account for between‐group differences (see Table 3 for details). (4) Estimation of Between‐Group Structure: The between‐group model also showed inadequate fit (*χ* ^2^/df = 3.027, RMSEA = 0.513, CFI = 0.790, TLI = 0.798, SRMR = 0.028), indicating that modeling the between‐group structure alone was insufficient and reinforcing the necessity of a unified MCFA approach (see Table 3 for details). The within‐group model captured individual‐level perceptual variation, resulting in unstable factor loadings, elevated RMSEA, and poor model fit. The between‐group model showed slightly higher CFI and TLI values than the within‐group model but still failed to meet acceptable fit criteria. It primarily reflected shared organizational perceptions while omitting within‐group individual variability. Estimating within‐group or between‐group covariance matrices separately was unable to represent the dual‐level variance structure of nested data, highlighting structural differences compared with MCFA. (5) MCFA: The final multilevel model, estimated using both within‐group and between‐group covariance matrices (Figure 4), showed excellent fit (*χ* ^2^/df = 1.211, RMSEA = 0.014, CF I = 0.978, TLI = 0.988, SRMR‐Within = 0.024, SRMR‐Between = 0.017), which confirms that the two‐factor structure of the adapted Chinese ORIC scale is well specified at the between‐group level, providing evidence for its structural validity at the organizational level (see Table 3 for details).

**TABLE 3:** The results of multilevel confirmatory factor analysis for the Chinese version of the adapted Chinese ORIC scale.

MCFA	Step T1		Step T3		Step T4	Step T5		
Level	Total		Within		Between	Within		Between
Factor	F1	F2	F1_W_	F2_W_	ORIC_B_	F1_W_	F2_W_	ORIC_B_
Item 1	0.639^∗∗∗^		0.996^∗∗∗^		0.624^∗∗∗^	0.607^∗∗∗^		0.806^∗∗∗^
	(0.022)		(0.001)		(0.025)	(0.022)		(0.026)

Item 2	0.614^∗∗∗^		0.993^∗∗∗^		0.598^∗∗∗^	0.773^∗∗∗^		0.777^∗∗∗^
	(0.028)		(0.002)		(0.026)	(0.017)		(0.027)

Item 3	0.610^∗∗∗^		0.994^∗∗∗^		0.591^∗∗∗^	0.667^∗∗∗^		0.744^∗∗∗^
	(0.027)		(0.002)		(0.026)	(0.021)		(0.027)

Item 4	0.701^∗∗∗^		0.994^∗∗∗^		0.688^∗∗∗^	0.732^∗∗∗^		0.832^∗∗∗^
	(0.022)		(0.001)		(0.023)	(0.018)		(0.026)

Item 5	0.592^∗∗∗^		0.998^∗∗∗^		0.574^∗∗∗^	0.709^∗∗∗^		0.735^∗∗∗^
	(0.027)		(0.001)		(0.026)	(0.019)		(0.027)

Item 6		0.562^∗∗∗^		0.988^∗∗∗^	0.537^∗∗∗^		0.746^∗∗∗^	0.690^∗∗∗^
		(0.028)		(0.003)	(0.029)		(0.016)	(0.028)

Item 7		0.641^∗∗∗^		0.997^∗∗∗^	0.623^∗∗∗^		0.768^∗∗∗^	0.772^∗∗∗^
		(0.027)		(0.001)	(0.028)		(0.015)	(0.028)

Item 8		0.623^∗∗∗^		0.994^∗∗∗^	0.601^∗∗∗^		0.794^∗∗∗^	0.730^∗∗∗^
		(0.032)		(0.002)	(0.028)		(0.014)	(0.029)

Item 9		0.529^∗∗∗^		1.000^∗∗∗^	0.505^∗∗∗^		0.645^∗∗∗^	0.671^∗∗∗^
		(0.032)		(0.000)	(0.030)		(0.020)	(0.029)

Item 10		0.556^∗∗∗^		0.995^∗∗∗^	0.533^∗∗∗^		0.764^∗∗∗^	0.718^∗∗∗^
		(0.028)		(0.001)	(0.030)		(0.016)	(0.029)

*Model Fit*								

LL	−13087.269		936.903		−9244.066	−13694.611		

AIC	27,282.796		−961.348		20,565.058	27,492.410		

BIC	27,488.661		−918.815		20,669.424	27,648.063		

*χ* ^2^/df	45.937		25.601		3.027	1.211		

RMSEA	0.137		0.663		0.513	0.014		

CFI	0.587		0.698		0.790	0.978		

TLI	0.530		0.600		0.798	0.988		

SRMR	0.571		0.035		0.028	—		

SRMR‐Within	—		—		—	0.024		

SRMR‐Between	—		—		—	0.017		

Group	1		1		56	56		

*N*	1120		1064		1120	1120		

*Note:* F = factor; W = within level; B = between level (e.g., F1 W denotes Factor 1 at the within level). “—” indicates that the value is not applicable or not estimated.^∗∗∗^ *p* < 0.001.

#### 3.6.2. Internal Consistency Reliability and Multilevel Retest Reliability

The internal consistency reliability and multilevel retest Reliability of the adapted Chinese ORIC scale was conducted using Sample B. The results showed that the total Cronbach’s α coefficient of the scale was 0.771 at the individual level and 0.883 at the organizational level. The Cronbach’s α coefficients of each factor ranged from 0.836 to 0.878 at the individual level and from 0.984 to 0.988 at the organizational level. The multilevel retest reliability coefficient was 0.756 (*p* < 0.001) at the individual level and 0.878 (*p* < 0.001) at the organizational level which indicates that both the internal consistency and multilevel retest reliability of the scale were higher at the organizational level than at the individual level.

## 4. Discussion

Within the context of ongoing healthcare reform, organizational members encounter adaptive challenges that are exacerbated by insufficient readiness, highlighting the need to assess and enhance ORC. This study undertook a rigorous cross‐cultural adaptation of the ORIC scale and evaluated its psychometric properties for use in the Chinese healthcare setting. This process resulted in a validated 10‐item, two‐factor Chinese ORIC scale, demonstrating strong suitability for measuring ORC in Chinese healthcare organizations.

To ensure appropriate interpretation of the results and methodological rigor in organizational‐level inference, it is necessary to clarify the sampling strategy and nested data structure. Sampling was conducted at the organizational level, with nurses nested within clinical organizations, which served as the primary clustering units. Participating organizations were selected from hospitals, and individual nurses were surveyed within each organization. This hierarchical structure reflects the organization of healthcare delivery in China, where clinical departments function as key units for staffing, management, and implementation of organizational change.

### 4.1. Scale Translation and Cross‐Cultural Adaptation

The translation and cross‐cultural adaptation of the original ORIC scale are essential first steps to ensure accurate understanding of its constructs in the target context and to support the validity and reliability of subsequent analyses [37]. The expert panel demonstrated excellent content validity (I‐CVI = 0.830–1.000; S‐CVI = 0.930), comparable to the original ORIC scale [20], with most items rated as highly relevant (mean scores > 4) and demonstrated good discriminative relevance across constructs. The content validity results exceeded established thresholds [38], further confirming the relevance and integrity of the Chinese version of the ORIC scale.

Additionally, the expert panel refined the instrument content by carefully evaluating the translation of the core term “change”, which has multiple possible Chinese expressions. To ensure semantic consistency, avoid ambiguity, and maintain conceptual equivalence, the research team reviewed dictionary definitions, relevant Chinese literature on ORC, and the ORIC application context [20, 39, 40]. Compared with “gǎi biàn”, which is more commonly associated with individual‐level adjustments, and “biàn gé”, which often refers to policy‐driven institutional reforms [39], the panel determined that “biàn gé” more accurately reflects the planned, collective, and systematic nature of organizational transformation assessed by the Chinese ORIC scale. This choice is also consistent with terminology commonly used in Chinese studies of organizational change and healthcare management [24, 40]. Furthermore, by examining items using the collective pronoun “we”, the expert panel situated individuals within the organizational context, thereby improving alignment between item wording and the intended construct. These adaptations ensured that the Chinese ORIC scale remained consistent with the original constructs while improving its applicability and reducing potential bias introduced by direct translation.

### 4.2. Psychometric Properties of the Adapted Chinese ORIC Scale

#### 4.2.1. Item Properties and Initial Factor Structure

EFA were conducted to examine item quality and explore the internal structure of the Chinese ORIC scale, providing a foundation for subsequent psychometric evaluation. All 10 items demonstrated satisfactory discriminability and representativeness. The extreme‐group comparison indicated statistically significant differences between high‐ and low‐scoring groups (*p* < 0.001), and the 95% confidence intervals for mean differences did not include zero, confirming effective item‐level discrimination.

The EFA results revealed a clear and stable two‐factor structure, with all items showing strong loadings (0.742–0.872) on their respective factors, supporting good construct validity and a distinct dimensional structure. The cumulative variance explained was 79.81%, exceeding the commonly accepted threshold and surpassing that of the original ORIC scale (69.60%) [20]. This higher explanatory power may reflect contextual characteristics of the Chinese healthcare setting. In particular, within hierarchical organizational structures and a collective decision‐making culture, change commitment may be interpreted as a shared organizational responsibility, whereas change efficacy may reflect confidence in collective rather than individual capability [41]. These contextual factors may have contributed to the increased variance explained while preserving the core construct meaning through culturally grounded interpretations.

Importantly, the Chinese version demonstrated a factor structure highly consistent with the original ORIC scale, with both change commitment and change efficacy clearly identified, providing strong evidence of cross‐cultural equivalence. Overall, the Chinese ORIC scale exhibited robust item properties and a well‐defined two‐factor structure, supporting its reliability and psychometric adequacy for use in Chinese healthcare settings.

#### 4.2.2. Validity Analysis

Recognizing the hierarchical structure of the data and the limitations of traditional CFA in handling nested data, we adopted the MCFA approach to validate the adapted Chinese ORIC scale. This method is well suited to organizational research, as it simultaneously models within‐group (individual‐level) and between‐group (organizational‐level) variance in nested data structures [42]. It is particularly important for examining multilevel structural validity, given that the ORIC instrument is completed by individuals but conceptualized at the collective level [42]. By explicitly accounting for clustering, MCFA reduces potential bias in parameter estimation and misinterpretation associated with single‐level CFA. Muthén’s MCFA framework has been widely applied in multilevel research across various fields, including team resilience [43], leadership [44], reliability estimation [43], and educational research [45].

From a practical perspective, nurses are nested within departments, where members typically share similar management practices, work environments, and experiences of organizational change [46]. In contrast, traditional CFA assumes independence of observations and ignores such clustering effects, which may lead to biased estimates and inadequate model fit [47]. MCFA overcomes this limitation by partitioning variance into within‐group and between‐group components, thereby providing a more appropriate framework for organizational‐level constructs in healthcare settings [43]. Specifically, within‐group variance reflects individual differences in perceptions within the same department, whereas between‐group variance captures systematic differences across departments, reflecting variation in leadership practices, organizational culture, and implementation climate.

The poor fit of the initial traditional CFA model (χ^2^/df = 45.937, RMSEA = 0.137, CFI = 0.587, TLI = 0.530, SRMR = 0.571) did not refute the existence of the two‐factor structure but rather revealed unaccounted variance due to the hierarchical nature of our data. From a data‐structure perspective, nurses are nested within clinical departments, violating the independence assumption of single‐level CFA. This is consistent with the cross‐cultural adaptation of the scale, in which nurses are conceptualized as organizational members responding from a collective rather than purely individual perspective. In addition, nurses within the same department are likely to share leadership practices, resource conditions, and implementation environments, resulting in correlated responses [48]. From a construct‐level perspective, the ORIC scale captures an organizational‐level construct reflecting shared cognition rather than individual attitudes [20]. Finally, in the Chinese healthcare context, hospital reforms are typically implemented through top‐down administrative systems, leading to substantial interdepartmental variation in implementation exposure and organizational climate [24]. This contextual heterogeneity likely reinforces clustering effects and reduces the appropriateness of single‐level CFA.

Overall, the poor fit of the initial CFA is better explained by hierarchical data structure, construct‐level misalignment, and contextual clustering effects, rather than by a deficiency of the two‐factor model. The ORIC scale is a multilevel construct reflecting shared perceptions of ORC rather than individual views [20]. While aggregation of individual responses requires sufficient within‐ and between‐group agreement for meaningful interpretation, the choice of multilevel CFA is primarily driven by the nested structure of the data and the violation of independence assumptions in single‐level CFA [34]. Traditional CFA may violate assumptions of independence and homogeneity, leading to biased estimates [33]. Therefore, MCFA was applied in this study. This methodological rationale is consistent with challenges reported by the original ORIC developers, who found that although a correlated two‐factor CFA model showed good fit, aggregation of individual responses to the organizational level required careful justification [20]. Their initial analyses indicated only moderate consistency for Change Commitment (ICC = 0.09) and low consistency for Change Efficacy (ICC = 0.02), suggesting limited support for direct aggregation [20]. However, using stronger interrater agreement indices such as within‐group agreement index (rWG) and Average Deviation from the Median (ADM), they demonstrated strong within‐group agreement for both dimensions (rWG(J) = 0.82), supporting aggregation [20]. While the original study used a phased approach in which data were aggregated before CFA [20], we applied MCFA to simultaneously examine within‐ and between‐group variance. Despite these methodological differences, both approaches supported a two‐factor structure of Change Commitment and Change Efficacy, with the original study reporting good model fit when factor correlations were allowed (CFI = 0.97, TLI = 0.96, SRMR = 0.05, RMSEA = 0.08) [20].

Previous studies in Western contexts have supported a stable two‐factor structure of the ORIC scale [17, 21, 22]. Building on this evidence, the present study further examined the generalizability of the factor structure in a Chinese healthcare context. In this study, EFA produced a cumulative explained variance of 79.81%, but comparisons with French, Australian, and South African versions are limited because not all studies reported this index [17, 21, 22]. The relatively more concentrated factor structure observed in this study may be related to differences in sampling composition and healthcare settings. Unlike prior validation studies that included heterogeneous samples from multiple levels of healthcare institutions [17, 21, 22], this study focused only on nurses in standardized tertiary hospitals with relatively uniform management systems and implementation contexts, which may have reduced response variability. In terms of measurement modeling, previous ORIC validation studies mainly used traditional CFA approaches [17, 21, 22]. By contrast, the present study incorporated the nested structure of organizational data by applying MCFA, allowing separation of within‐ and between‐group variance. The final model showed good fit indices, providing additional support for the robustness of the two‐factor structure when hierarchical data characteristics are properly addressed.

Overall, these findings are consistent with the two‐dimensional structure of the ORIC scale and further suggest that sampling characteristics and analytical approaches may influence variance distribution and factor analytic results across studies. Future research should therefore prioritize multilevel modeling approaches when validating organizational‐level constructs in cross‐cultural settings to ensure methodological rigor and enhance comparability across studies.

#### 4.2.3. Reliability Analysis

Reliability assessment is essential for establishing the psychometric properties of a scale, as it ensures stability and consistency across different cultural contexts [35]. In this study, the internal consistency and test–retest reliability of the adapted Chinese ORIC scale were evaluated using a multilevel approach, examining its consistency over time and across organizational levels. Results demonstrated that the overall scale, its two factors, and test–retest reliability all met acceptable standards [32, 49].

The multilevel test–retest reliability assessed after a 2 week interval remained stable, confirming the adapted Chinese ORIC scale’s stability across measurement occasions [32]. Compared with the original version, the adapted Chinese ORIC scale demonstrated higher internal consistency in both the “Change Commitment” and “Change Efficacy” factors [20]. This improvement can be largely attributed to better alignment with the working environment and cultural context of Chinese nurses. “Change Commitment” places greater emphasis on employees’ collective willingness for change [20]. Within the context of China’s ongoing healthcare reform, where nurses routinely work in a change‐oriented environment, this emphasis may enhance internal consistency [50]. Similarly, “Change Efficacy” focuses on nurses’ collective confidence in change outcomes [20], aligning with Chinese nurses’ adaptability to systemic reforms, which in turn enhances this dimension’s reliability [51]. Furthermore, internal consistency was higher at the organizational level than at the individual level in this study which aligns with the original design [20]. ORC primarily reflects shared values among members within the same institution, which tend to be relatively homogeneous, thereby fostering higher internal consistency [7].

Taken together, MCFA results supported the stability of the two‐factor structure of the Chinese ORIC scale and provided evidence for its structural validity in this context. The model also showed good fit after accounting for the hierarchical nature of the data, indicating its suitability for organizational‐level use. Acceptable internal consistency and stable test–retest reliability further suggest that the Chinese ORIC scale is a reliable and stable tool for assessing organizational ORC. However, the sampling strategy and organizational context should be interpreted within the broader structure of the Chinese healthcare system. Tertiary Grade A hospitals in China represent the most advanced level of healthcare institutions, characterized by standardized management systems, strong implementation capacity, and frequent exposure to organizational innovation. Therefore, while this enhances measurement stability and internal consistency, it may also limit variability and should be considered when generalizing findings to lower‐level hospitals or more heterogeneous healthcare settings.

### 4.3. Practical Value of the Chinese ORIC Scale

Organizations across various sectors, including healthcare, face growing pressures to implement rapid and continuous change, underscoring the need for reliable instruments to assess their readiness to undertake such change [52, 53]. This study focused on the cross‐cultural adaptation and validation of the ORIC scale among Chinese nurses. As the largest professional group within the healthcare system, nurses are not only key performers of change policies and technological innovations but also a core constituency that directly experiences the effects of change and contributes critically to its success [54]. Amid ongoing strategic investments in nursing education, research, and leadership, nurses are increasingly taking on roles as clinical experts, educators, researchers, and policy leaders [55]. This evolving professional landscape requires nurses to continuously prepare for emerging challenges, such as complex disease burdens, aging populations, digital integration, inclusive leadership, and the translation of global evidence into practice [55].

Within this context, the observed pattern indicates that participants generally expressed a high level of readiness for change. However, the lower level of agreement on the Change Efficacy dimension compared with Change Commitment suggests that, although nurses were largely willing to support and engage in organizational change, they expressed comparatively less confidence in shared implementation capacity and perceived organizational support. This discrepancy is consistent with the conceptual distinction underlying the ORIC framework, which posits that commitment and efficacy represent related yet distinct components of organizational readiness [20]. Within the context of ongoing healthcare reforms, nurses may readily endorse the value and necessity of change while simultaneously experiencing uncertainty regarding resource availability and collective implementation capability [20].

Importantly, the Chinese ORIC scale enables healthcare administrators and nursing managers to objectively evaluate ORC and formulate targeted implementation strategies. Specifically, they can use the scale before implementing major initiatives (e.g., digital health transformation, quality improvement programs, clinical pathways, or policy reforms) to assess departmental readiness. For instance, some departments may show high change commitment but lower change efficacy, suggesting willingness to change but insufficient confidence in resources, knowledge, leadership support, or implementation capacity. In these cases, managers can prioritize targeted training, resource allocation, mentoring, and implementation support rather than focusing only on motivation. Furthermore, the scale can be used longitudinally to monitor readiness across different stages of implementation. Repeated assessments can help identify declines in readiness, emerging barriers, and the effectiveness of support interventions. Departments with persistently low readiness may need additional communication, leadership engagement, or organizational support to ensure successful implementation. At the organizational level, aggregated ORIC results may also inform strategic decision‐making. By comparing readiness profiles across departments, healthcare organizations can allocate implementation resources more efficiently, tailor communication strategies to local needs, and prioritize interventions in units facing greater implementation challenges. Therefore, the Chinese ORIC scale can serve not only as a valid measurement instrument but also as a practical resource to support organizational change initiatives. Overall, it provides a solid foundation for assessing and enhancing ORC among staff in healthcare organizations and for guiding future efforts to strengthen their capacity to respond to and engage in ongoing healthcare transformation.

### 4.4. Limitations

This study has several limitations that should be acknowledged. First, the use of a nonprobability sampling strategy in tertiary Grade A hospitals may introduce selection bias and limit the generalizability of the findings to healthcare settings with different resource levels and organizational characteristics. Second, data were collected using an online self‐administered questionnaire, which may also introduce selection bias, as nurses who were more available or more willing to participate in organizational change–related research were more likely to respond. Third, although a pilot test was conducted; cognitive interviews were not included in the cross‐cultural adaptation process, which may have limited the depth of cultural adaptation and content validity. Fourth, constraints related to study design and field data collection prevented a full examination of convergent validity, discriminant validity, and criterion‐related validity. Nevertheless, the present study provides evidence supporting content validity, structural validity, and reliability, with particular emphasis on multilevel structural validity. Finally, the study focused exclusively on nurses, which may limit the applicability of the findings to other healthcare professional groups. Overall, these limitations should be considered when interpreting the findings, and future studies are encouraged to adopt more robust methodological designs, including longitudinal validation to examine temporal stability, inclusion of diverse healthcare professional groups to enhance external validity, and incorporation of objective outcome indicators to better evaluate the effectiveness of organizational change in practice.

## 5. Conclusions

The Chinese version of the ORIC scale exhibits strong psychometric properties across all validation steps, demonstrating robust reliability and validity. These findings enhance the understanding of the scale’s cross‐cultural applicability, particularly in contexts beyond Western cultures. Looking ahead, healthcare organizations can utilize the validated Chinese ORIC scale to routinely assess employees’ readiness for change. This will support the development of targeted interventions, strengthen organizational preparedness during change initiatives, facilitate the management of employee expectations, improve the effectiveness of change implementation, and inform the design of more robust change strategies.

## Author Contributions

Yu Tian: conceptualization, methodology, investigation, data curation, formal analysis, writing–original draft, and writing–review and editing. Ling Yu: conceptualization, methodology, supervision, project administration, writing–review and editing, and correspondence. Hui Wang: methodology, validation, supervision, and writing–review and editing. Yanze Shi: investigation and data curation. Lei Liu: investigation and resources. Yingjie Wang: investigation and resources. Zihan Li: data curation and validation.

## Funding

This work was supported by the Chinese Nursing Association (ZHKYQ202301).

## Disclosure

All authors read and approved the final manuscript.

## Conflicts of Interest

The authors declare no conflicts of interest.

## Supporting Information

Additional supporting information can be found online in the Supporting Information section.

## Supporting information


**Supporting Information** Supporting Appendix. The supporting appendix provides detailed information supporting the translation and cross‐cultural adaptation of the Chinese version of the ORIC scale. Specifically, it includes the characteristics of the translation team, the complete translation and adaptation process, and the expert content validity evaluation with corresponding revision records.

## Data Availability

The data that support the findings of this study are available on request from the corresponding author. The data are not publicly available due to privacy or ethical restrictions.
