# Supplementary material for: Cross‐Cultural Adaptation and Validation of the Organizational Readiness for Implementing Change (ORIC) Scale Among Nurses in Chinese Healthcare Institutions: A Multilevel Psychometric Assessment
Source: J Nurs Manag. 2026 Aug 12;2026:2913316. doi: 10.1155/jonm/2913316 (PMC13469762; doi:10.1155/jonm/2913316)
Supplement: Supplementary file 1 [file JONM-2026-2913316-s001.docx]

### **Appendix Table 1. The basic characteristics of the translation team (n = 8)**

| Expert | Role | Degree | Professional Title | Professional Background | Research Theme Background | International  educational experience | Professional experience (years) |
| --- | --- | --- | --- | --- | --- | --- | --- |
| F1 | Forward Translator | PhD | Associate Chief Physician | Implementation Science | Yes | Yes | 15 |
| F2 | Forward Translator | Master | Associate Professor | English Linguistics | No | Yes | 15 |
| R1 | Proofreader | PhD | Associate Professor | Implementation Science | Yes | Yes | 10 |
| R2 | Proofreader | Master | Chief Nurse | Nursing Management | Yes | No | 20 |
| B1 | Back Translator | Master | Associate Professor | English Linguistics | No | Yes | 13 |
| B2 | Back Translator | Master | Associate Professor | English Linguistics | No | Yes | 16 |
| R3 | Proofreader | PhD | Professor | Implementation Science | Yes | Yes | 11 |
| R4 | Proofreader | Master | Chief Nurse | Nursing Management | Yes | No | 18 |

Note: F1-F2 indicate the experts involved in forward translation; R1-R4 indicate the review experts; B1-B2 indicate the experts involved in back-translation.

**Appendix Table 2. Process of the ORIC Scale Translation and Cross-cultural Adaptation**

| Items of the original ORIC | Translation process of the ORIC scale |
| --- | --- |
| 1. Are committed to implementing this change. | F1: We are committed to implementing this change.  F2: We are committed to implementing this change.  R1&R2: We are committed to implementing this change.  B1: We are committed to implementing this change.  B2: We are dedicated to implementing this change.  R3&R4: We are committed to implementing this change. |
| 2. Are determined to implement this change. | F1: We are determined to implement this change.  F2: We are determined to implement this change.  R1&R2：We are determined to implement this change.  B1: We are determined to implement this change.  B2: We have the determination to implement this change.  R3&R4: We have the determination to implement this change. |
| 3. Are motivated to implement this change | F1: We are motivated to implement this change.  F2: We are motivated to implement this change.  R1&R2: We are motivated to implement this change.  B1: We are motivated to implement this change.  B2: We are enthusiastic about implementing this change.  R1&R2: We have the motivation to implement this change. |
| 4. Will do whatever it takes to implement this change | F1: We will make every effort to implement this change.  F2: We will spare no effort in implementing this change.  R1&R2: We will go all out to implement this change.  B1: We are adequately committed to implementing this change.  B2: We are determined to implement this change.  R3&R4: We will make every effort to implement this change. |
| 5. Want to implement this change | F1: We want to implement this change.  F2: We intend to implement this change.  R1&R2: We intend to implement this change.  B1: We want to implementing this change.  B2：We plan to implement this change.  R3&R4: We intend to implement this change. |
| 6. Feel confident that they can manage the politics of implementing this change | A1: The employees are confident in addressing political issues during the implementation of changes.  A2：We are confident in the to effectively manage the policies required to implement this change.  R1&R2: We believe that we are capable of effectively managing the relevant policies involved in the implementation of change.  B1: We trust that we are able to effectively manage the policies associated with the change implementation.  B2：We believe that we are capable of effectively handling the policies involved in implementing the change.  R3&R4: We believe that we have the ability to effectively implement change-related policies. |
| 7. Feel confident that the organization can support people as they adjust to this change | A1: The employees believe that the organization can support people in adapting to this change.  A2: We have confidence that this organization can help us adapt to this change.  R1&R2: We believe that the organization can provide support as employees adapt to this change.  B1: We believe the organization can support staff as they adapt to this change implementation.  B2: We believe the organization will support employees as they adjust to the change implementation.  R3&R4: We believe that the organization will provide support as employees adapt to change. |
| 8. Feel confident that they can coordinate tasks so that implementation goes smoothly | A1: The employees are confident in their ability to coordinate tasks to ensure the smooth implementation of change.  A2：We are confident in the ability to coordinate task progress and ensure smooth implementation.  R1&R2: We believe that we are capable of coordinating various tasks to ensure smooth implementation.  B1: We trust that we are able to coordinate tasks to ensure a smooth implementation.  B2: We believe that we are capable of managing tasks to keep the implementation on track.  R3&R4: We believe that we have the ability to coordinate various tasks to ensure smooth implementation. |
| 9. Feel confident that they can keep track of progress in implementing this change | A1: The employees are confident in tracking the progress of change implementation.  A2: We are confident in the ability to track progress in implementing this change.  R1&R2: We believe that we can keep up with the progress of implementing the change.  B1: We trust that we are able to keep track of the progress of implementing the change.  B2: We believe that we are capable of keeping pace with the implementation of the change.  R3&R4: We believe that we have the ability to manage the progress of implementing the change. |
| 10. Feel confident that they can handle the challenges might arise in implementing this change | A1: The employees are confident in their ability to handle potential challenges during the change process.  A2：We are confident in the ability to handle any challenges that may arise during the implementation of this change.  R1&R2: We believe that we are capable of handling any challenges that may arise during the process of implementing change.  B1: We trust that we are able to cope with any challenges that may arise during the implementation of the change.  B2: We believe that we are capable of handling any potential challenges during the change implementation.  R3&R4: We believe that we have the ability to address the challenges encountered during change implementation. |

Note: F1-F2 indicate the experts involved in forward translation; R1-R4 indicate the review experts; B1-B2 indicate the experts involved in back-translation. A portion of the table content is presented in Chinese to reflect the cross-cultural adaptation process of the ORIC scale and to respect the linguistic and cultural context in which it is applied.

Appendix Table 3. Content validity results of the Chinese equivalent of the original ORIC scale

| Item | Number of experts assigning a rating ≥ 3 | I-CVI | Experts’ comments and suggestions | Adoption of the expert suggestion (Yes/No) | Revised final item |
| --- | --- | --- | --- | --- | --- |
| We are committed to implementing this change. | 5 | 0.83 | It is suggested that the behavior implied by “being committed to” be specified more clearly; replacing it with “to fully promote” would be more appropriate for assessing the change commitment dimension. | yes | We are fully promoting this change |
| We have the determination to implement this change. | 6 | 1 | The item is clearly worded with no apparent issues and is therefore recommended to be retained. |  | We have the determination to implement this change. |
| We have the motivation to implement this change. | 6 | 1 | The item is clearly worded with no apparent issues and is therefore recommended to be retained. |  | We have the motivation to implement this change. |
| We will make every effort to implement this change. | 6 | 1 | The item is clearly worded with no apparent issues and is therefore recommended to be retained. |  | We will make every effort to implement this change. |
| We intend to implement this change. | 6 | 1 | The item is clearly worded with no apparent issues and is therefore recommended to be retained. |  | We intend to implement this change. |
| We believe that we have the ability to effectively implement change-related policies. | 6 | 1 | The item is clearly worded with no apparent issues and is therefore recommended to be retained. |  | We believe that we have the ability to effectively implement change-related policies. |
| We believe that the organization will provide support as employees adapt to change. | 5 | 0.83 | It is recommended that “providing support” be specified as “providing resources or training support” to enhance measurability. | Yes | We believe that the organization will provide resources or training support as employees adapt to change. |
| We believe that we have the ability to coordinate various tasks to ensure smooth implementation. | 6 | 1 | The item is clearly worded with no apparent issues and is therefore recommended to be retained. |  | We believe that we have the ability to coordinate various tasks to ensure smooth implementation. |
| We believe that we have the ability to manage the progress of implementing the change. | 6 | 1 | The item is clearly worded with no apparent issues and is therefore recommended to be retained. |  | We believe that we have the ability to manage the progress of implementing the change. |
| We believe that we have the ability to address the challenges encountered during change implementation. | 6 | 1 | The item is clearly worded with no apparent issues and is therefore recommended to be retained. |  | We believe that we have the ability to address the challenges encountered during change implementation. |

Note: A portion of the table content is presented in Chinese to reflect the cross-cultural adaptation process of the ORIC scale and to respect the linguistic and cultural context in which it is applied.
